# Identification of Cofilin-1 Induces G0/G1 Arrest and Autophagy in Angiotensin-(1-7)-treated Human Aortic Endothelial Cells from iTRAQ Quantitative Proteomics

**DOI:** 10.1038/srep35372

**Published:** 2016-10-17

**Authors:** Huang-Joe Wang, Sung-Fang Chen, Wan-Yu Lo

**Affiliations:** 1 School of Medicine, China Medical University, No. 91, Hsueh-Shih Road, Taichung, Taiwan 40402, R.O.C; 2 Division of Cardiovascular Medicine, Department of Medicine, China Medical University Hospital, No. 2, Yude Road, Taichung Taiwan 40447, R.O.C; 3 Cardiovascular Research Laboratory, China Medical University Hospital, No. 2, Yude Road, Taichung Taiwan 40447, R.O.C; 4 Department of Chemistry, National Taiwan Normal University, No. 88, Sec. 4, Ting-Chow Rd, Taipei, Taiwan 11677, R.O.C; 5 Cardiovascular & Translational Medicine Laboratory, Department of Biotechnology, Hung Kuang University, No. 1018, Sec. 6, Taiwan Boulevard, Shalu District, Taichung Taiwan 43302, R.O.C

## Abstract

The angiotensin-converting enzyme 2/angiotensin-(1-7)/Mas axis is a pathway that acts against the detrimental effects of the renin-angiotensin system. However, the effects of angiotensin-(1-7) on endothelial protein expression and the related phenotypes are unclear. We performed a duplicate of iTRAQ quantitative proteomic analysis on human aortic endothelial cells (HAECs) treated with angiotensin-(1-7) for 6 hours. Cofilin-1 was identified as a highly abundant candidate with consistent >30% coverage and >1.2-fold overexpression in the angiotensin-(1-7)-treated group. Gene ontology analysis showed that the “regulation_of_mitosis” was significantly altered, and cell cycle analysis indicated that the 6-hour angiotensin-(1-7) treatment significantly induced G0/G1 arrest. Knockdown of the *cofilin-1 (CFL1*) gene suggested the G0/G1 phase arrest was mediated by the modulation of p27 and the p21/Cyclin/CDK complex by Cofilin-1. Interestingly, quiescent HAECs escaped G0/G1 arrest upon angiotensin-(1-7) treatment for 24 hours, and angiotensin-(1-7) induced autophagy by upregulating Beclin-1 and microtubule-associated protein 1 light chain 3b-II expression, which was also attenuated by A779 pre-treatment and *CFL1* knockdown. After pre-treatment with 3-methyladenine (3MA), treatment with angiotensin-(1-7) for 24 h induced significant G0/G1 phase arrest and apoptosis, suggesting a pro-survival role of autophagy in this context. In conclusion, Cofilin-1 plays a dominant role in angiotensin-(1-7)-induced G0/G1 arrest and autophagy to maintain cellular homeostasis in HAECs.

The angiotensin-converting enzyme 2 (ACE2)/angiotensin-(1-7)/Mas axis is a well-known counter-regulatory pathway in the renin-angiotensin system (RAS)^1^. In this axis, angiotensin-(1-7) is produced from angiotensin I or angiotensin II via the catalytic activity of ACE2, an ACE homologue, and the human plasma concentrations of immunoreactive angiotensin-(1-7) are reported to be 1.0–9.5 pmol/L^2^. There is a body of evidence for the endothelial protective effects of the ACE2/angiotensin-(1-7)/Mas receptor axis. This axis is a recently discovered pathway that can reverse the effects of Angiotensin II in a number of tissues, mainly by inhibiting the cell growth, migration and inflammation that occurs as a result of Angiotensin II activity, preventing adverse remodeling and the subsequent dysfunction of the cardiovascular system^1^,^3^,^4^,^5^,^6^,^7^. Chronic angiotensin-(1-7) infusion was also indicated to improve renal endothelial function by increasing endogenous nitric oxide in apolipoprotein E-deficient mice^8^. In contrast, the knockout of the angiotensin-(1-7) Mas receptor causes endothelial dysfunction in C57Bl/6 mice^9^. Recently, we also reported that angiotensin-(1-7) treatment could significantly attenuate glycated albumin-induced endothelial interleukin-6 production^10^. Taken together, these results suggest that the amplification of ACE2/angiotensin-(1-7)/Mas provides protection against the development of endothelial dysfunction. However, the dominant impact of acute angiotensin-(1-7) treatment on endothelial cells remains unclear.

Quantitative proteomics is an important branch of proteomics that is used to quantify and identify all the proteins expressed by a genome or in a complex mixture. Isobaric tags for relative and absolute quantification (iTRAQ) were developed in 2004 by Ross *et al*. This unique approach labels samples with four independent isobaric tags with the same mass and, upon fragmentation in tandem mass spectrometry (MS/MS), the technique produces four unique reporter ions (m/z from 114 to 117) that provide quantitative information based on the integration of the peak areas of the four different samples^11^. The advanced combination of proteomics and bioinformatics provides a convenient opportunity to study primary changes in the global proteome, such as the molecular expression levels in cells at any time point or for any treatment. With bioinformatics development, there are more studies that use gel-free quantitative proteomic platforms to investigate the variable and complex physiological processes^12^,^13^,^14^.

Cofilin-1 is a 19-kDa protein that is ubiquitously present in eukaryotes and promotes actin filament dynamics during motility, development and cytokinesis^15^. Cofilin-1 is a member of the ADF/Cofilin family. This family has three members: Cofilin-1 (CFL1, non-muscle cofilin), Cofilin-2 (CFL2, muscle cofilin) and ADF (destrin)^16^. Cofilin-1 is the major protein, and its regulation has been reported to be associated with various types of cancers^17^,^18^,^19^. In 2013, Yang *et al*. also suggested Cofilin-1 is a new biomarker for poor prognosis of gallbladder cancer^20^. However, whether the expression levels of Cofilin-1 are important for the regulation of the cell cycle and autophagy progression in human aortic endothelial cells (HAECs) is still unclear.

We used iTRAQ analysis to quantitatively profile the protein expression of HAECs in response to treatment with angiotensin-(1-7) for 6 h. The protein Cofilin-1 was identified as a unique candidate from the duplicated iTRAQ analysis that displayed a higher coverage (>30%) and consistent overexpression (>1.2-fold) in angiotensin-(1-7)-treated endothelial cells than in the untreated cells. Based on a series of molecular biological validations, Cofilin-1 was identified as a regulator of cell cycle arrest, autophagy and apoptosis in HAECs for the first time. This study advances our understanding the role of Cofilin-1 in HAEC homeostasis under the ACE2/angiotensin-(1-7)/Mas axis.

## Results

A schematic flowchart is presented in Supplemental data 1. The study began through a quantitative proteomics study for novel biomarkers involving in angiotensin-(1-7) treated HAECs.

### Identification of the different protein expression levels between HAECs treated with or without angiotensin-(1-7) for 6 h and GO analysis

**Figure 1:**
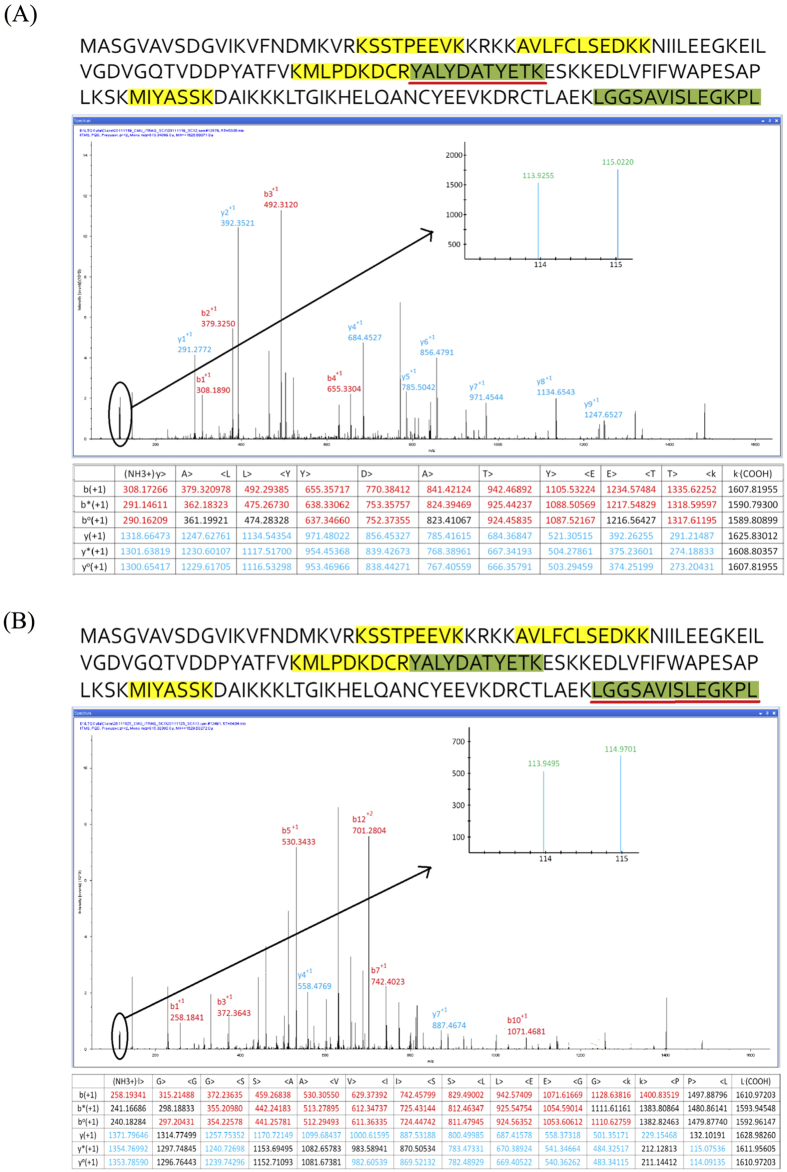
A representative MS/MS spectrum of the corresponding amino acid sequences (**A**) YALYDATYETK and (**B**) LGGSAVISLEGKPL used in the identification and quantitation of Cofilin-1 from duplicated iTRAQ analysis. The complete peptide sequences are listed. Peptides with high confidence (*p* < 0.01) are labeled in green, and the peptides with medium confidence (p < 0.05) are labeled in yellow. The relative intensity of the iTRAQ reporter ions and associated b and y ions used to identify and quantify the relative protein expression for each subject were generated by Proteome Discoverer (Thermo Scientific, version 1.1) with the search algorithm Mascot.

The iTRAQ experiments were performed as duplicate independent experiments. There were 6,469 and 9,675 MS/MS spectra identified, which led to the identification of 1,791 and 2,111 unique peptides with ≥34 ion scores, respectively. With the false discovery rate of <3% taken into account, 590 and 648 proteins were identified from the independent duplicate iTRAQ analyses (#1119 and #1128), respectively. There were 590 and 648 proteins quantified in the two biological replicates, respectively. The proteins were quantified using the iTRAQ ratios by assigning at least 2 MS/MS spectra from the duplicate iTRAQ experiments to distinct iTRAQ reporter ion signals (Supplemental data 2A,B, iTRAQ data). In this study, Cofilin-1 was identified as a unique candidate that had consistent (>30%) coverage and >1.2-fold overexpression in the angiotensin-(1-7)-treated group compared with the untreated group (Control) from the duplicate iTRAQ analysis (Supplemental data 3). The representative MS/MS spectra of the corresponding amino acid sequences YALYDATYETK (Fig. 1A) and LGGSAVISLEGKPL (Fig. 1B) were used in the identification and quantitation of the Cofilin-1 protein from databanks of duplicate iTRAQ analysis.

Among the #1119 and #1128 iTRAQ analyses, proteins that were overexpressed >1.2-fold in the angiotensin-(1-7)-treated group were screened for GO annotation. In respect to the GO database, the differentially expressed proteins were divided into three categories: molecular function (MF), biological processes (BP) and cellular components (CC) (supplemental data 4). Among of the categories, the ‘regulation_of_mitosis’ of the BP component exhibited a significant difference (*p* < 0.05) between the control and 6-h angiotensin-(1-7)-treated groups, suggesting that angiotensin-(1-7) may affect the cell cycle regulation of HAECs.

### The gene and protein levels of cofilin-1 were upregulated by angiotensin-(1-7) and attenuated by A779

**Figure 2:**
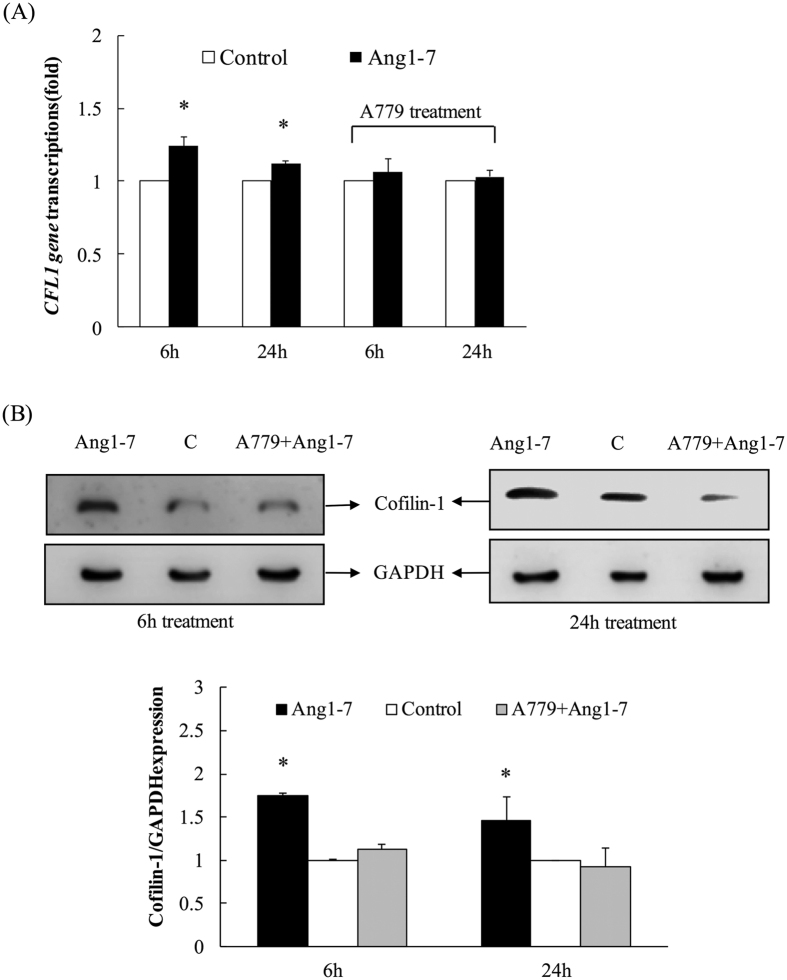
The target *CFL1* and protein quantifications were validated. (**A**) The dynamic results revealed that *CFL1* was significantly upregulated in angiotensin-(1-7)-treated HAECs and attenuated by the Mas-receptor antagonist A779. (**B**) The expression of Cofilin-1 increased significantly in the groups treated with angiotensin for 6 h or 24 h and was also attenuated by A779 pre-treatment. The data are expressed as the mean ± SEM for three independent experiments. *p < 0.05 indicates a significant difference relative to the control (n = 5).

The dynamic results revealed that the cofilin-1 gene (*CFL1*) was significantly upregulated in angiotensin-(1-7)-treated HAECs, and this upregulation was attenuated by treatment with the Mas-receptor antagonist, A779 (Fig. 2A). Compared with the control group, the expression of the *CFL1* gene in the groups treated with angiotensin-(1-7) for 6 h or 24 h increased by an average of 1.25-fold and 1.18-fold, respectively. The expression of the Cofilin-1 protein increased by an average of 1.75-fold and 1.36-fold in the groups treated with angiotensin-(1-7) for 6 h or 24 h compared with the control group, respectively (Fig. 2B). The upregulation of the *CFL1* gene and the protein expression in the angiotensin-(1-7)-treated groups were attenuated to similar levels as the control by A779 pre-treatment.

### Angiotensin-(1-7) induced cell cycle arrest at the G0/G1 phase and the attenuation of cell cycle arrest by A779 and *CFL1* siRNA

**Figure 3:**
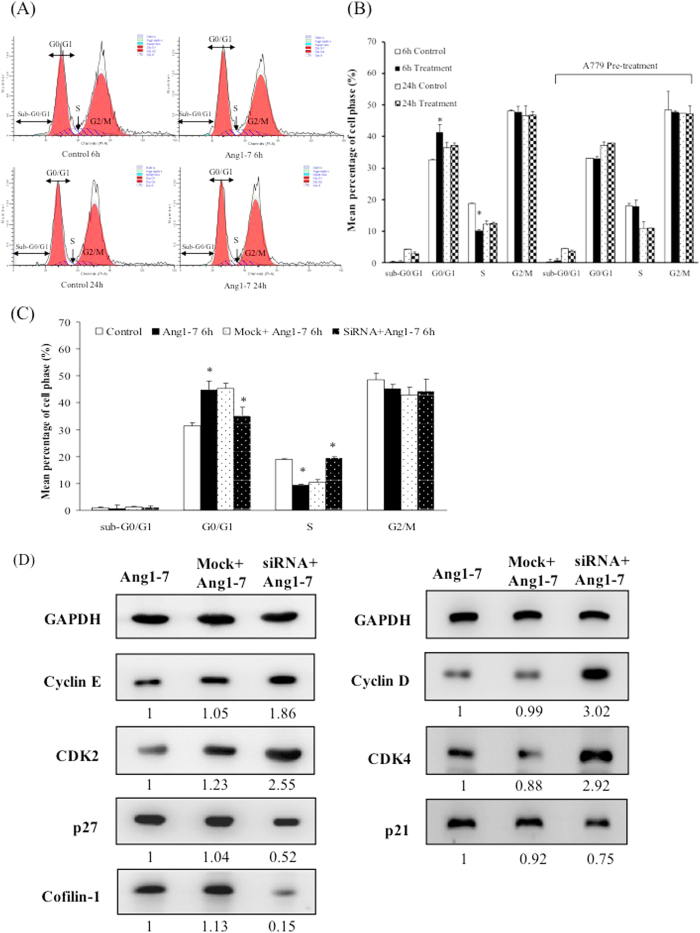
The cell cycle regulation induced by angiotensin-(1-7). (**A**) After treatment with angiotensin-(1-7) for 6 h or 24 h, the HAECs were analyzed by flow cytometry. The X- and Y-axes are intensity of the PI staining and cell number, respectively. The data represent the distributions of the cells in the G0/G1, S, and G2/M phases. (**B**) Angiotensin-(1-7) treatment for 6 h induced G0/G1 phase arrest and decreased the number of cells in S phase; the G0/G1 arrest was attenuated by A779 pre-treatment. The data are expressed as the mean ± S.E.M. (n = 5) (**C**) The G0/G1 arrest were also attenuated by *CFL1* siRNA pre-treatment. The data are expressed as the mean ± SEM (n = 5). (**D**) The cells were harvested for total protein determination, and the expression levels of p21, p27, CDK2, CDK4, CyclinD, CyclinE and Cofilin-1 were determined via western blots. The mock transfection was used as the control, and similar data were obtained from three independent experiments. *p < 0.05 indicates a significant difference relative to the 6-h control group.

Based on the result from the GO analysis, we evaluated the regulation of the cell cycle upon angiotensin-(1-7) treatment. HAECs treated with angiotensin-(1-7) for 6 h exhibited a significant increase in the arrest at the G0/G1 phase and a decrease in the proportion of cells in S phase (Fig. 3A). In response to angiotensin-(1-7) treatment, the percentage of G0/G1 phase cells significantly increased from 31.6% to 40.3%, and the S-phase cells decreased from 18.7% to 10.2%. These results suggest that treatment with angiotensin-(1-7) for 6 h reduces DNA synthesis and induces G0/G1 phase arrest in HAECs; however, these same alterations were not observed after 24 h (Fig. 3B). The percentage of G0/G1 phase cells was significantly reversed from 40.3% to 33.8% upon angiotensin-(1-7) treatment for 6 h with A779 pretreatment (Fig. 3B). These results demonstrate that the significant G0/G1 arrest can also be attenuated by A779 pre-treatment.

In 2009, Tsai *et al*. reported that the knockdown of the *CFL1* gene expression can reduce the number of cells in the G1 phase and is associated with p27 expression in lung cancer^19^. To explore the mechanisms underlying Cofilin-1 regulation, we performed specific siRNA transfection to knockdown the *CFL1* gene. The cell cycle and related proteins in the HAECs after treatment with angiotensin-(1-7) for 6 h were evaluated. The results demonstrated that the percentage of cells in the G0/G1 phase generated with angiotensin-(1-7) treatment (44.8%, Ang1-7 group) was attenuated by *CFL1* siRNA transfection (34.1%, siRNA + Ang1-7 group) and that the reduction in the percentage of cells in the S phase with angiotensin-(1-7) treatment (8.6%, Ang1-7 group) was also recovered (19.2%, siRNA + Ang1-7 group) (Fig. 3C). Furthermore, the *CFL1* siRNA transfection not only reduced the protein expression of Cofilin-1 but also downregulated p27 and p21. In contrast, the siRNA transfection upregulated Cyclin D, Cyclin E, CDK2, CDK4 and CDK relative to the mock transfected (control) group (Fig. 3D). These data also suggest that angiotensin-(1-7)-induced Cofilin-1 overexpression regulates G0/G1 phase arrest via p27 and the p21/Cyclin/CDK complex.

### The effect of angiotensin-(1-7) on the autophagic activity in HAECs

**Figure 4:**
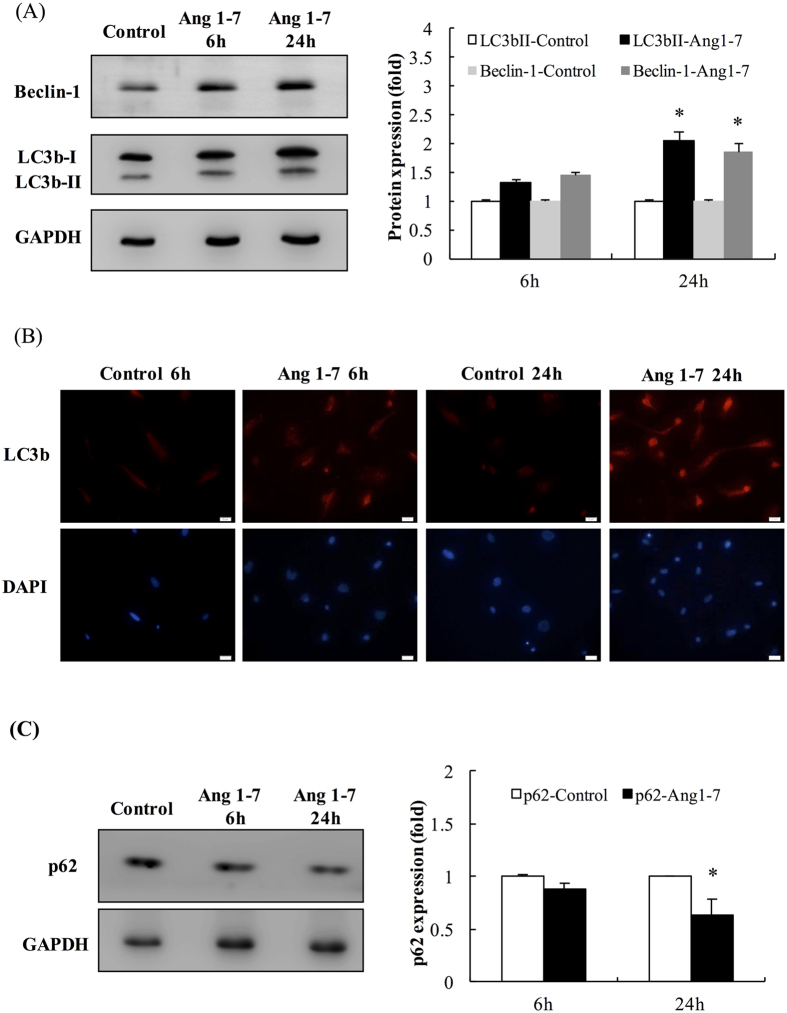
The effects of angiotensin-(1-7) on autophagic markers in serum-starved HAECs. (**A**) Left: The expression levels of Beclin-1, LC3b-I and LC3b-II. Right: The protein expression levels for Beclin-1, LC3b-I and LC3b-II were quantified in the different groups. (**B**) The cellular localization of LC3b in HAECs. LC3-II immunoreactivity in the group treated with angiotensin-(1-7) for 24 h was stronger than in the group treated for 6 h. LC3b-II is indicated in red, and DAPI is indicated in blue. (Scale bar: 10 μm). (**C**) Left: The expression levels of p62. Right: The expression levels of the protein p62 were quantified in the different groups. The data are expressed as the mean ± SEM (n = 5). *p < 0.05 indicates a significant difference relative to individual control groups.

To study the differential regulation of the cell cycle in the groups treated with angiotensin-(1-7) for 6 h or 24 h, we analyzed LC3b-II expression. LC3b-II is derived from LC3-I after autophagy induction and is incorporated into the growing autophagosomes; thus, LC3b-II is a good indicator of autophagic activity^21^. Moreover, we also detected the levels of Beclin-1, an important factor involved in the recruitment of membranes to form autophagosomes^22^. In Fig. 4A, the expression levels of LC3b-II and Beclin-1 were significantly elevated by 2.15-fold and 1.86-fold, respectively, in the group treated with angiotensin-(1-7) for 24 h compared with the control. Because LC3b-II exhibited higher efficiency than Beclin-1 in this experiment, the LC3b-II levels were used for further confirmation in an immunofluorescence assay in this study (Fig. 4B). The intercellular expression of p62 was significantly reduced by 36% in the group treated with angiotensin-(1-7) for 24 h compared with the control group (Fig. 4C). These results suggest that angiotensin-(1-7) induced a time-dependent upregulation in autophagic activity via LC3b-II and Beclin-1. Thus, we used the 24-h treatment to perform the advanced studies.

### Autophagy was induced by angiotensin-(1-7) and attenuated by A779 and *CFL1* siRNA transfection

**Figure 5:**
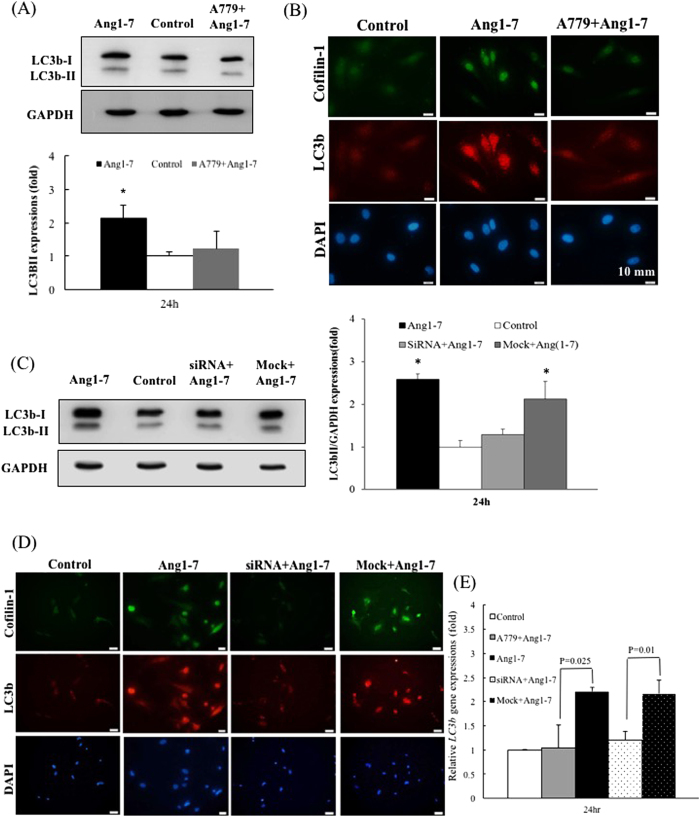
The effects of A779 and *CFL1* siRNA pre-treatments on the autophagic activity in HAECs treated with angiotensin-(1-7) for 24 h. (**A**) The expression levels of LC3b-I and LC3b-II in the control group and the groups pretreated with or without A779. (**B**) The immunoreactivity of Cofilin-1 and LC3b in HAECs treated with angiotensin-(1-7) for 24 h was stronger than in the control groups, whereas pre-treatment with A779 abolished this effect. (**C**) The expression levels of, LC3b-I and LC3b-II in the control group, the group that was treated with angiotensin-(1-7) for 24 h, the group that was pretreated with *CFL* siRNA transfection and then treated with angiotensin-(1-7) for 24 h and the group that was pretreated with the mock treatment and then with angiotensin-(1-7) for 24 h. (**D**) The immunoreactivity of Cofilin-1 and LC3b-II in HAECs treated with angiotensin-(1-7) for 24 h was stronger than in the control HAECs, whereas pre-treatment with *CFL* siRNA abolished the effects (Scale bar: 50 μm). All the above slides were double-stained for Cofilin-1 (green) and LC3b (red), and DAPI nucleic acid staining is shown in blue. (**E**) In the above 5 different groups, the gene expressions levels of LC3b as indicated by the qPCR were quantified. The data are expressed as the mean ± SEM (n = 5). *p < 0.05 indicates a significant difference relative to the individual control groups.

Pre-treatment with A779 reversed the angiotensin-(1-7)-induced LC3b-II expression from 2.27-fold to 1.12-fold compared with the control group (Fig. 5A), and this result was further corroborated by an immunofluorescence assay (Fig. 5B). Additionally, the role of Cofilin-1 in the autophagic effects was verified by siRNA transfection. As demonstrated in Fig. 5C, pre-treatment with Cofilin-1 siRNA for 24 h can prevent the angiotensin-(1-7)-induced increasing in the expression level of LC3b-II (LC3b-II level from 2.74-fold to 1.32-fold). This result was also supported by an immunofluorescence assay (Fig. 5D). The related LC3b-II gene expression levels in the A779 and siRNA treatments were shown in Fig. 5E. Taken together, the data suggest that angiotensin-(1-7) treatment for 24 hr induced autophagy via Cofilin-1 overexpression.

### The effect of angiotensin-(1-7)-induced autophagy on the cell cycle phase and proliferation of HAECs

**Figure 6:**
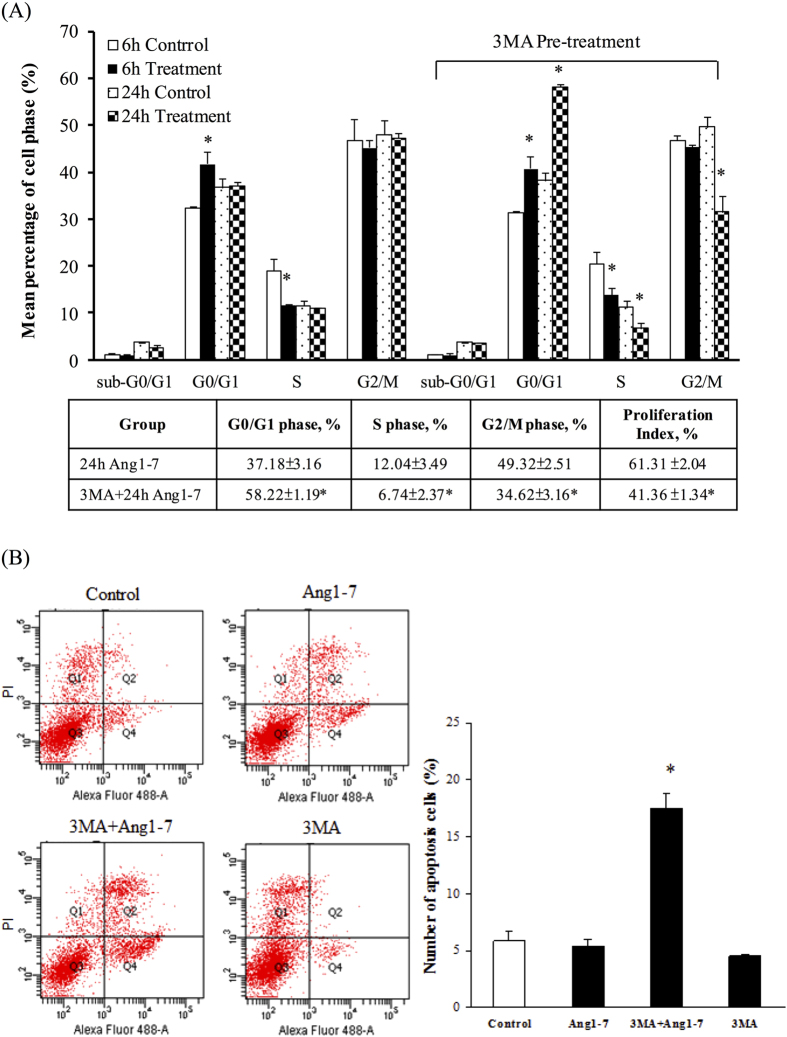
The effect of 3MA pretreatment on cell cycle arrest, proliferation and apoptosis in angiotensin-(1-7)-treated HAECs. (**A**) Pre-treatment with 3MA before treatment with angiotensin-(1-7) for 24 h significantly induced G0/G1 phase arrest and decreased the number of cells in the S phase in a time-dependent manner. The cell percentages of each phase after 24-h angiotensin-(1-7) treatment are shown in the below table. The total percentage of cells in the S and G2/M phases represents the proliferation index (n = 5). (**B**) Pre-treatment with 3MA before treatment with angiotensin-(1-7) for 24 h increased the apoptotic level of HAECs by almost 3-fold. **Left:** The typical quadrant diagrams of the flow cytometric analysis of apoptotic cells stained with FITC-AnnexinV and PI. The Q1, Q2 and Q4 quadrants represent necrotic cells, late apoptotic cells and early apoptotic cells, respectively. **Light:** The quantification of the number of apoptotic cells (Q2 + Q4) from the different groups. Compared with the group treated with angiotensin-(1-7) for 24 h only (6.3%), the number of apoptotic cells was significantly increased in the 3M-A + Ang1-7 group (17.52%). The data are expressed as the mean ± SEM (n = 5). *p < 0.05 indicates a significant difference relative to the angiotensin-(1-7)-only group.

Angiotensin-(1-7) treatment for 6 h induced G0/G1 phase arrest (from 33.16% to 41.75%), whereas treatment for 24 h did not induce G0/G1 arrest (from 37.25% to 38.54%) (Fig. 6A). 3MA is an inhibitor of class III PI3K that prevents the formation of autophagosomes^23^. In the 3MA pre-treated group, angiotensin-(1-7) significantly upregulated G0/G1 phase arrest (from 37.18% to 58.22%) and downregulated the number of cells in the S and G2/M phases (from 12.04% to 6.74% and 49.3% to 34.62%, respectively) after 24 h of angiotensin-(1-7) treatment. These results demonstrate that the angiotensin-(1-7)-induced G0/G1 phase arrest can be attenuated by autophagy in HAECs treated for 24 h. Among the 3 groups (6-h angiotensin-(1-7), 3MA + 6-h angiotensin-(1-7) and 24-h angiotensin-(1-7)), there were no significant differences in the proliferation indices (data not shown). However, the proliferation index was significantly downregulated in the 3MA + 24-h angiotensin-(1-7) group (41.36 ± 1.34%) compared with the group treated with angiotensin-(1-7) for 24 h (61.31 ± 2.04%) (Table below Fig. 6A). These results demonstrate that the inhibition of the autophagic activity (3MA pre-treatment) can markedly downregulate the proliferation index in HAECs treated with angiotensin-(1-7) for 24 h.

To determine whether the downregulation of cell proliferation was due to apoptosis or necrosis, we probed the mode of cell death (Fig. 6B). The number of apoptotic cells in serum-starved HAECs treated with 3MA + angiotensin-(1-7) for 24 h was significantly increased by 11.2% (Q2 + Q4, from 6.3% to 17.5%) compared with the angiotensin-(1-7) group; however, the number of necrotic cells (Q1) was not altered. These data demonstrate that the inhibition of autophagic activity (3MA pre-treatment) can markedly upregulate apoptosis by 2.8-fold in HAECs treated with angiotensin-(1-7) for 24 h. Taken together, treatment with angiotensin-(1-7) + 3MA for not only 6 h but also 24 h, induced G0/G1 phase arrest and apoptosis. However, this induction can be overcome by angiotensin-(1-7)-induced autophagy at a later time point.

## Discussion

In our previous hyperglycemia study, we found that treatment with angiotensin-(1-7) for 6 h could significantly attenuate HAEC inflammation^10^. Therefore, we used a 6-h treatment to compare different protein expression levels at the beginning of this study. Then, we collected the proteins with >1.2-fold overexpression from the angiotensin-(1-7)-treated group for GO analyses to review the potential effects. The “regulation_of_mitosis” component was identified as a notable indicator. On the other hand, Cofilin-1 was identified from the duplicated iTRAQ analysis as the unique high abundance candidate with consistent >30% coverage and >1.2-fold overexpression in HAECs treated with angiotensin-(1-7) for 6 h. Therefore, we hypothesized that the “regulation_of_mitosis” maybe regulated by Cofilin-1 overexpression and then initiated a novel pharmacological study based on Cofilin-1.

Evidence that angiotensin-(1-7) is produced in the vasculature was first discovered 24 years ago^24^. The renin-angiotensin system (RAS) is thought to be a circulating endocrine system with octapeptide angiotensin (Ang) II as an effector hormone and angiotensin-(1-7) as an inactive fragment^1^. Many of the cardiovascular effects of angiotensin-(1-7) are completely blocked by the antagonist A779, which suggests that these effects are mediated by a receptor that is sensitive to A779^1^,^25^. Among the experiments, A779 pre-treatment was also shown to significantly attenuate Cofilin-1 overexpression (Fig. 2), G_0_/G_1_ phase arrest (Fig. 3B) and autophagy (Fig. 5A) in angiotensin-(1-7)-treated HAECs. This important finding is the first report that angiotensin-(1-7) can induce Cofilin-1 expression, G_0_/G_1_ phase arrest and autophagy via the MAS receptor.

On the other hand, siRNA transfection not only knocked down the *CFL1* gene but also significantly inhibited the expression of the cell cycle-related p21 and p27 proteins, increasing Cyclin D, Cyclin E, CDK2 and CDK4 expression (Fig. 3D). In 2009, Tsai *et al*. reported that Cofilin-1 can regulate the expression of p27 and is important for G0/G1 phase progression. Moreover, we demonstrated that the knockdown of Cofilin-1 caused p27 and partial p21 downregulation and rescued the G_0_/G_1_ phase arrest in HAECs treated with angiotensin-(1-7) for 6 h, which is consistent with the observations of Tsai *et al*. This study is also the first to indicate that angiotensin-(1-7) can regulate G0/G1 arrest via the modulation of Cofilin-1 and the Cyclin/CDK/CDK inhibitor complexes.

Interestingly, we observed angiotensin-(1-7) treatment for 6 h induced G0/G1 phase arrest and significantly reduced the number of cells in S phase, but the arrest was not maintained after 24 h of treatment (Fig. 3D). Furthermore, we did not observe significant changes in cell proliferation in the groups treated with angiotensin-(1-7) for 6 h or 24 h (data not shown). Thus, we investigated whether autophagy was involved in cell cycle regulation. As a quality control mechanism of cytoplasmic proteins, autophagy plays important roles in a variety of human diseases, including neurodegenerative diseases, cancer, cardiovascular disease, and infectious and inflammatory diseases^26^. The microtubule- associated protein 1 light chain 3 (LC3b) and Beclin-1 are the major elements in the formation of autophagosomes^27^. A basal level of autophagy helps to maintain cellular homeostasis. Conversely, unrestrained and excessive autophagic activity can lead to variable programmed cell death and is considered a contributing factor to carcinogenesis, cardiovascular and neurodegenerative disorders^27^,^28^,^29^,^30^. In a previous study, *in vitro* experiments have demonstrated that the autophagy of endothelial cells can promote angiogenesis^31^, the complete function of autophagic activity in endothelial cells still remains elusive.

In this study, we provide the first evidence that autophagic activity can be induced in HAECs treated with angiotensin-(1-7) for 24 h (Fig. 4) and the induction of autophagy can be attenuated by pre-treatment with A779 and *CFL1* siRNA transfection, suggesting that the Mas receptor and *CFL1*-dependent pathways are involved in this regulation (Fig. 5). Angiotensin-(1-7), the main component of the ACE2/angiotensin-(1-7)/Mas axis, causes beneficial effects on the central nervous system. However, some studies have investigated the effect of angiotensin-(1-7) on autophagy that indicated that angiotensin-(1-7) works against Angiotensin II-induced cardiomyocyte autophagy and it inhibits autophagy in the brain of rats with hypertension; however, the mechanism is currently unclear^32^,^33^. We also observed another special phenotype in this study, i.e., when autophagy was inhibited by 3MA pre-treatment, the G0/G1 arrest and the apoptotic activity of serum-starved HAECs increased and cell proliferation decreased in the group treated with angiotensin-(1-7) for 24 h (Fig. 6). Obviously, autophagy plays an important role in maintaining normal cellar homeostasis by restoring the cell cycle in angiotensin-(1-7)-treated HAECs. In previous studies, significant evidence has shown that autophagy can regulate cell proliferation and decrease the number of cells in G0/G1 phase arrest^34^,^35^. These studies suggested that autophagy is most active in the G0/G1 phases of the cell cycle. Others studies have also suggested that autophagy plays an important role in the proliferation of stem/progenitor cells under hypoxic stress conditions and in ventricular hypertrophy, as it helps maintain cellular homeostasis^36^,^37^. In conclusion, autophagy seems to be critical for regulating cell growth and promoting cell survival in times of stress.

Moreover, 3MA (autophagy inhibitor) pre-treatment also significantly enhanced G0/G1 arrest and reduced cell proliferation in angiotensin-(1-7)-treated HAECs in a time-dependent manner, demonstrating that autophagy rescued 24 h cell survival through G0/G1 arrest recovery (Fig. 6A). 3MA pre-treatment reduced cell proliferation through apoptosis in 24-h angiotensin-(1-7)-treated HAECs (Fig. 6B). Current data has suggested that autophagy reduces DNA damage and apoptosis, possibly by removing damaged mitochondria and protein^38^. A previous study suggested that angiogenesis-(1-7) induces apoptosis in circulating fibrocytes^39^. In contrast, angiogenesis-(1-7) was also demonstrated to overcome apoptosis in angiotensin II-induced alveolar epithelial cells and human umbilical vein endothelial cells via the Mas receptor^40^,^41^. The variable effects of angiogenesis-(1-7) appear to be dependent on the variable cell types.

**Figure 7:**
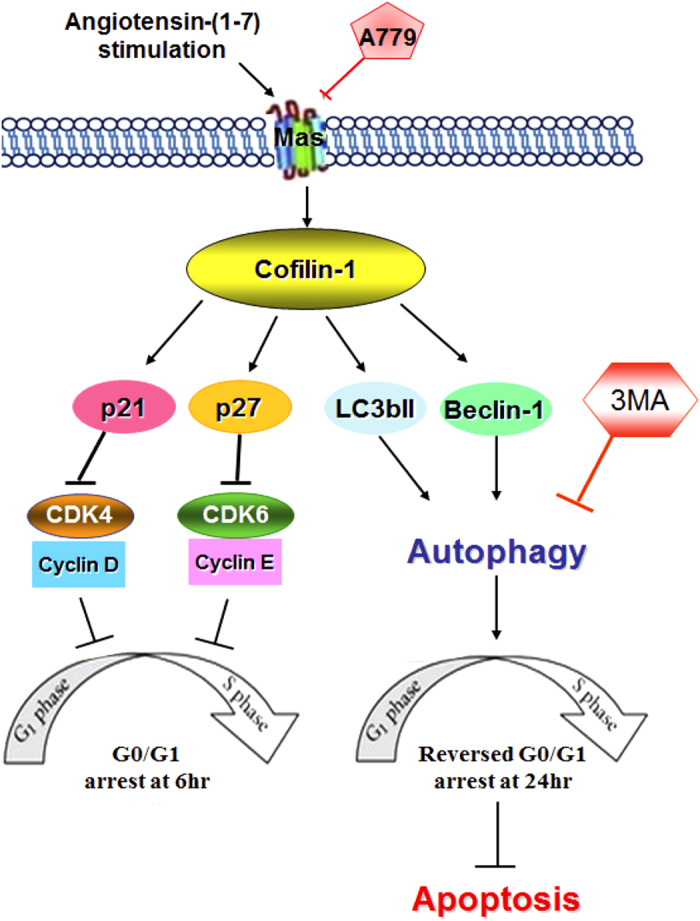
The proposed model for the mechanism of angiotensin-(1-7)-induced Cofilin-1 in the regulation of G_0_/G_1_ phase arrest and autophagy in serum-starved HAECs.

In conclusion, this study demonstrated that angiotensin-(1-7) induces cofilin-1 expression in serum-starved HAECs. The induction of Cofilin-1 mediates acute G0/G1 arrest and autophagy to inhibit apoptosis (keep cell proliferation) during angiotensin (1-7) treatment (Fig. 7). These findings suggest that Cofilin-1 plays an important pro-survival role in quiescent HAECs upon angiotensin-(1-7) exposure and represents a novel modulator that maintains cellular homeostasis in RAS-related cardiovascular pathophysiology. On the other hand, the unstable proliferation of HAECs is a crucial step for induced angiogenesis. This novel pathway regulated by Cofilin-1 also provides a new revision in the angiogenesis research of RAS-related physiology.

## Material and Methods

### Cell culture and sample treatments for iTRAQ

HAEC and endothelial cell growth medium were purchased from Cell Applications, Inc. (Cell Applications, Inc., San Diego, CA). The HAECs were seeded in 6-well plates at 5 × 10^5^ cells/well and grown overnight under serum-starved conditions in M199 medium, then supplemented with 1% fetal bovine serum before stimulation. The cells were treated with or without 100 nM angiotensin-(1-7) (Sigma–Aldrich, St. Louis, MO) for 6 h and harvested by centrifugation at 300 × g for 15 min. The pellets were washed with ice-cold PBS twice and resuspended in RIPA buffer (Sigma-Aldrich, St. Louis, MO) to extract the total proteins. The total proteins were enriched using a 3-kDa centrifugal filter as described by the manufacturer (Millipore, Merck KGaA, Germany). This process was repeated twice using ddH_2_O for desalting. The amount of protein in each concentrated/desalted sample was determined via a Bradford protein assay (Bio-Rad, Hercules, CA), and the samples were stored at −80 °C for subsequent processing. We performed biological duplicate experiments to monitor the consistency of the iTRAQ results.

### Chemicals and reagents for iTRAQ

Chromatography grade acetonitrile from Merck KGaA was used. Modified porcine trypsin (sequencing grade) was obtained from Promega (Madison, WI). The iTRAQ 4-plex reagent kits were purchased from Applied Biosystems (Framingham, MA). Desalting spin columns were purchased from Pierce (Thermo Scientific, San Jose, CA). The column packing materials for the analytical column were purchased from MACHEREY-NAGEL (Düren, Germany). The packing materials for the trap column were purchased from Michrom Bioresources (Auburn, CA). All other chemicals and reagents were of analytical grade and purchased from Sigma-Aldrich (St. Louis, MO), unless otherwise stated.

### Reduction, alkylation, digestion, and labeling with iTRAQ

The protein samples were reduced, alkylated, digested, and labeled with iTRAQ reagents according to the recommended protocol (Applied Biosystems, Framingham, MA) and our pervious study^14^. The protein pellets were resuspended in 0.5 M triethylammonium bicarbonate (TEAB) at pH 8.5 with 2% sodium dodecyl sulfate (SDS) and reduced with 50 mM Tris (2-carboxyethyl) phosphine (TCEP) for 1 h at 60 °C. Then, the sample was alkylated with 200 mM s-methyl methanethiosulfonate (MMTS) at room temperature for 10 min. A total of 70 μg of protein was digested overnight in a tryptic solution (30/1, w/w) at 37 °C. The digested samples were labeled with the iTRAQ reagents as follows: 114 for the controls (without angiotensin-(1-7)) and 115 for the treated samples (with angiotensin-(1-7)). After 2 h of iTRAQ labeling, the samples were mixed and dried via centrifugal evaporation. We performed a duplicate of iTRAQ quantitative proteomic analysis to monitor the consistency of the results.

### Strong cation exchange chromatography (SCX) and LC-ESI-MS/MS analysis

Peptide separation was performed using a Polysulfoethyl A Column (200 mm L × 2.1 mm i.d., 5 μm, 300 Å, PolyLC, Columbia, MD) with an Agilent 1100 binary HPLC (Agilent Technologies, Wilmington, DE) over a 90 min gradient. Eighty fractions were collected after SCX fractionation and then pooled into 24 fractions. The fractions were purified using C-18 Spin columns (Thermo Scientific, San Jose, CA) for further nano-LC-ESI-MS/MS analysis^14^.

### Database search and iTRAQ quantification

Protein identification and quantification were performed using the search algorithm Mascot (V2.3.2) and the Protein Discoverer software (ThermoFisher, version 1.1). The search was performed against the SwissProt v.2012_08 database (531,473 sequences) using the following search parameters: taxonomy, *Homo sapiens*; enzyme, trypsin; max. miss cleavages, 1; fixed modifications, methylthiolation N-terminal iTRAQ 4plex, lysine iTRAQ 4plex; variable modifications, methionine oxidation, tyrosine iTRAQ 4plex; MS peptide tolerance, 1.5 Da; and MS/MS tolerance, 0.6 Da. Protein identifications were only accepted when ≥2 spectra exhibited ion scores above 35 (95% confidence).

### Data analysis

The relative protein expression was determined based on the ratio of the reporter ions for the peptides (115:114). Proteins with sequence coverage of >30% and a fold-change cutoff ratio of >1.2-fold were selected as biomarkers in the angiotensin-(1-7)-treated group. The relative intensity of the iTRAQ reporter ions and the associated b and y ions were used to identify and quantify the relative protein expression. Moreover, the molecular function (MF), the cellular component (CC) and the biological process (BP) of the selected proteins were annotated using the Gene Ontology (GO) database (2012_12), and the networks were ranked (Phalanx Biotech Group, Inc.).

### Studies of angiotensin-(1-7) inhibition by the Mas-receptor antagonist

A779 (Bachem) is a Mas-receptor antagonist of angiotensin-(1-7). The HAECs were pre-treated with or without A779 (1 μM) for 10 min before exposure to 10 nM angiotensin-(1-7) for 6 or 24 h.

### Quantitative analysis of gene expression

Real-time quantitative polymerase chain reaction (real-time Q-PCR) was performed according our previous study^42^. The primers used for real-time quantitative PCR are listed below:


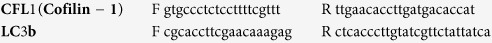


### Western blots

The HAECs of the different groups were washed once with 1 × PBS and lysed with 50 μL RIPA buffer (Sigma-Aldrich) supplemented with 1% protease inhibitor cocktail (Roche, Mannheim, Germany). Equal amounts of the protein extracts (35 μg) were loaded, separated by 15% SDS-PAGE, and blotted onto a polyvinylidene fluoride membrane. The membranes were then incubated overnight with the anti-LC3b (1:1,000), anti-Beclin-1 (1:1,000) (Cell Signaling Technology, USA), anti-CyclinD (1:1,000), anti-CyclinE (1:1,000), anti-CDK4 (1:1,000), anti-CDK2 (1:500), anti-p27 (1:100), anti-Cofilin-1 (1:2,000) and anti-GAPDH (1:2,000) (Santa Cruz Biotechnology, Santa Cruz, CA) antibodies. The immunostaining was visualized using enhanced chemiluminescence (Pierce, Rockford, USA).

### Analysis of cell cycle and proliferation by flow cytometry

After treatment with A779 and angiotensin-(1-7) as described previously, HAECs (10^6^ cells) were fixed in 75% ethanol at 4 °C overnight. The cell pellets were resuspended in propidium iodide (PI) and subjected to flow cytometric analysis after centrifugation. The DNA content was determined by flow cytometry with an argon laser excited at 488 nm (BD FACSCanto™ system, Becton Dickinson Corp, San Jose, CA). The DNA histograms, including the DNA quantification, were analyzed. The total percentage of cells in the S and G2/M phases was defined as the proliferation index. The synchronization of the cells was performed using the method described by Olszewska *et al*.^43^.

### *CFL1* gene silencing

The HAECs were transfected with 100 nM ON-TARGETplus SMARTpool Human *CFL1* siRNA (Dharmacon, Thermo Scientific Dharmacon, Lafayette, CO) for 24 h, and the mock control groups was treated with the reagent without the *CFL1* siRNA. After angiotensin-(1-7) treatment, the mRNA and protein expression levels, the cell cycle phase, the double immunofluorescence staining and the amount of apoptosis were examined.

### Double immunofluorescence staining

The cells were grown on glass coverslips using the protocols described above. The cell monolayers were fixed in neutral formalin (pH 7.2) at 4 °C for 3 hour. After washing in PBS, the samples were permeated with methanol for 30 min at −20 °C. Subsequently, the coverslips were blocked in PBS/5% bovine serum albumin (BSA) for 30 min. The fixed cells were then incubated with an anti-LC3B antibody (#2775, Cell Signaling Technology, 1:400 dilution) at 4 °C overnight. Next, the samples were washed three times with PBS and incubated with the Dylight-549 goat anti-rabbit IgG secondary antibody (Jackson Immunoresearch, West Baltimore Pike, PA, 1:500) at room temperature for 1 hour. Then, they were washed several times with PBS. After the first staining with anti-LC3B, the samples were incubated with an anti-Cofilin 1 antibody (sc-53934, Santa Cruz Biotechnology, Santa Cruz, CA, 1:50 dilution) at 4 °C overnight. The samples were washed with PBS and incubated with the Dylight-488 goat anti-mouse IgG secondary antibody (Jackson Immunoresearch, 1:250 dilution) at room temperature for 1 hour. Then, the samples were washed several times with PBS. The coverslips were mounted on slides using approximately 50 μl of mounting medium with 4′, 6-diamidino-2-phenylindole (DAPI) (SouthernBiotech, Birmingham, AL, USA). Cells were visualized with a fluorescence microscope (BX50, Olympus) with an Olympus DP72 CCD camera.

### Inhibition of angiotensin-(1-7)-induced autophagy by 3-methyladenine (3MA)

3MA is a known specific inhibitor of autophagic sequestration^44^. In the group pre-treated with 3MA, the cells were pre-treated with 5 mM of 3MA for 1 h before angiotensin-(1-7) treatment.

### Analysis of apoptosis

The cells were divided into four groups: control, angiotensin-(1-7) only, 3MA + angiotensin-(1-7) and 3MA only. The cells were harvested and incubated with 1 g/ml FITC-AnnexinV and 1 g/ml PI (BD Biosciences, CA) for 10 min. The apoptotic cell numbers were analyzed by flow cytometry (BD FACSCanto™ system, Becton Dickinson Corp., San Jose, CA). The ratios of early apoptotic, late apoptotic, necrotic and viable cells were calculated with the following formula: the percent (%) = (the number of early apoptotic, late apoptotic, necrotic cells and viable cells)/the total cell number^45^. The experiment was repeated three times.

### Statistical analysis

Independent experiments were conducted to assess the significant difference between the control and other experimental groups. Significant differences were determined using Student’s *t*-test and defined as *p* < 0.05.

## Additional Information

**How to cite this article**: Wang, H.-J. *et al*. Identification of Cofilin-1 Induces G0/G1 Arrest and Autophagy in Angiotensin-(1-7)-treated Human Aortic Endothelial Cells from iTRAQ Quantitative Proteomics. *Sci. Rep.* **6**, 35372; doi: 10.1038/srep35372 (2016).

## Supplementary Material

Supplementary data 1

Supplement data 2A

Supplement data 2B
