# Supplementary material for: Identification of Cofilin-1 Induces G0/G1 Arrest and Autophagy in Angiotensin-(1-7)-treated Human Aortic Endothelial Cells from iTRAQ Quantitative Proteomics
Source: Sci Rep. 2016 Oct 17;6:35372. doi: 10.1038/srep35372 (PMC5066316; doi:10.1038/srep35372)
Supplement: Supplementary data 1 [file srep35372-s1.pdf]

Identification of Cofilin-1 Induces G0/G1 Arrest and Autophagy in Angiotensin-(1-7)-treated Human Aortic Endothelial Cells from iTRAQ Quantitative Proteomics

Huang-Joe Wang[1,2,3], Sung-Fang Chen[4*], Wan-Yu Lo[5]*

Supplemental data 1 Experimental flowchart. The HAECs were stimulated with 100 nM Angiotensin-(1-7) for 6 h, untreated cells as the control. Proteins and their related pathways were identified via iTRAQ labeling, quantitative proteomic analysis and GO analysis. The regulations of cell cycle and autophagy were identified via *CFL1* gene knockdown and A779 pretreatments.

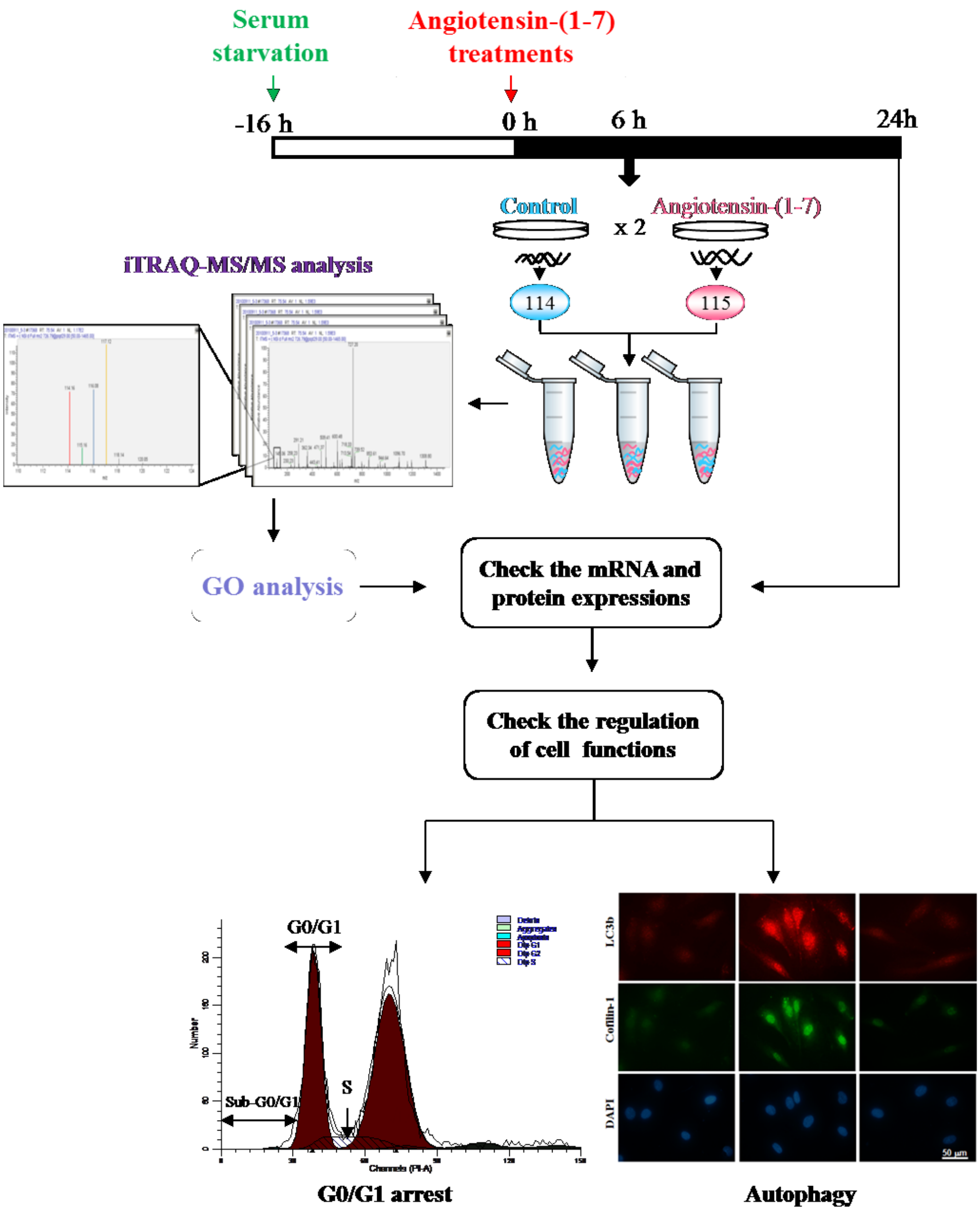

Supplemental data 3 The Cofilin-1 protein is the unique candidate had the higher coverage (>30%) and displayed consistent overexpression (>1.2 fold) in the angiotensin-(1-7) treated cells in comparison with the control from the duplicated iTRAQ analysis.

**iTRAQ analysis.**	**Accession NO.**	**Coverage [%]**	**Match peptides**	**115/114 ratio**	**Function**	**Gene name & locations**
#1119	P23528	36.14	30	1.3	Binds to F-actin and exhibits pH-sensitive F-actin depolymerizing activity. Regulation of actin cytoskeleton dynamics. Important for normal progress through mitosis and normal cytokinesis. Plays a role in the regulation of cell morphology and cytoskeletal organization	Cofilin 1 (Non-Muscle) *11q13.1*
#1128	P23528	30.52	36	1.2		

Supplemental data 4 GO annotation of the overexpressed proteins (> 1.2-fold overexpression in the angiotensin-(1-7)-treated group). The proteins were divided into three categories: molecular function (MF), biological process (BP) and cellular component (CC). Left: The top 5 components for MF, BP, CC of the differentially expressed proteins according to GO database are shown. Right: Each enumerated annotation is assigned by the enrichment score represented as *P value* (differentially expressed level).

(A)

**Component name (Molecular function)**	***P* value**
ACTIN_FILAMENT_BINDING	0.214
MICROTUBULE_BINDING	0.358
PROTEIN_TYROSINE_KINASE_ACTIVITY	0.407
PROTEIN_KINASE_INHIBITOR_ACTIVITY	0.412
KINASE_INHIBITOR_ACTIVITY	0.468

(B)

**Component name (Biology process)**	***P* value**
REGULATION_OF_MITOSIS	0.0152 *
S_PHASE_OF_MITOTIC_CELL_CYCLE	0.0471
CELLULAR_RESPONSE_TO_STIMULUS	0.0549

POSITIVE_REGULATION_OF_MAPKKK_CASCADE	0.0715
POSITIVE_REGULATION_OF_CELL_CYCLE	0.118

(C)

**Component name (Cellular component)**	***P* value**
MICROTUBULE	0.247
SPINDLE_MICROTUBULE	0.313
MICROTUBULE_ASSOCIATED_COMPLEX	0.382
MICROTUBULE_ORGANIZING_CENTER	0.402
ACTIN_CYTOSKELETON	0.412
